# 2552. Comparative Pharmacodynamic Evaluation of Commonly Utilized, Oral, 1^st^ - 3^rd^ Generation Cephalosporins against *S. aureus* and *E. coli* in the Mouse Thigh Infection Model

**DOI:** 10.1093/ofid/ofad500.2169

**Published:** 2023-11-27

**Authors:** Alexander Lepak, Sujata M Bhavnani, Catharine Vincent, Brian D VanScoy, Helio S Sader, David Andes

**Affiliations:** University of Wisconsin School of Medicine and Public Health, Madison, Wisconsin; Institute for Clinical Pharmacodynamics, Schenectady, NY; Institute for Clinical Pharmacodynamics, Schenectady, NY; Institute for Clinical Pharmacodynamics, Schenectady, NY; JMI Laboratories, North Liberty, Iowa; University of Wisconsin, Madison, Wisconsin

## Abstract

**Background:**

Cephalexin (LEX), cefuroxime axetil (CXM), and cefpodoxime (CPD) are common oral cephalosporins used to treat gram-positive and gram-negative infections. However, there is a paucity of PK/PD analyses to inform optimal dosing regimens, which drug may be preferred for a particular organism, and the susceptibility testing interpretive criteria (STIC) that may apply to help clinicians in their selection.

**Methods:**

5 *E. coli* (EC) and 5 *S. aureus* (SA) clinical strains were used. MICs were determined by CLSI methods. Pharmacokinetics of LEX, CXM, and CPD were performed in mice at 10, 40, 160, and 320 mg/kg. Dose-ranging efficacy studies were performed against all strains (dose range 2.5-320 mg/kg/4h). Treatment outcome was determined by organism burden in the thighs (CFU) at the end of each experiment (24 h). The dose-response (D-R) data was analyzed using the E_max_ Hill equation. Data was fit to the PK/PD index time above MIC (T >MIC) for free drug concentrations. Static and cidal target exposures were calculated for each strain, and targets compared by One Way ANOVA.

**Results:**

EC MIC ranges: LEX 4-64mg/L, CXM 1-8 mg/L, CPD 0.125-8 mg/L. SA MIC ranges: LEX 2-16 mg/L, CXM 0.5-2 mg/L, CPD 2-8 mg/L. All three drugs performed similarly well against SA with relatively steep D-R curves and achieving >1-log kill against 5 of 5 strains. For EC studies, LEX demonstrated a flat, more muted effect with only 2 of 5 strains achieving a 1-log kill. Comparatively, CXM and CPD demonstrated a steeper, more potent D-R curve against EC with a >1-log kill against 4 of 5 strains for CXM and 5 of 5 for CPD. The PK/PD index T >MIC fit the treatment efficacy data well (R^2^ 0.66-0.91). Summary statistics for PK/PD analyses are shown in **Figure**.

**Figure:**
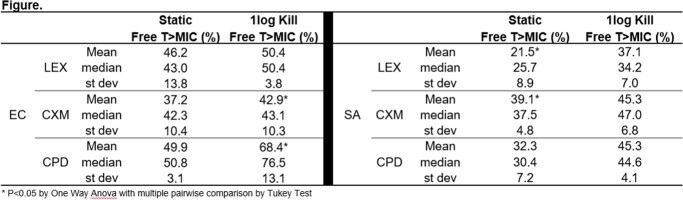

**Conclusion:**

Oral 1^st^-3^rd^ generation cephalosporins exhibited efficacy against SA and EC in the mouse thigh infection model. Stasis and 1-log kill targets for the three drugs against SA were 25-35% and 35-45% T >MIC, respectively. Lower targets were noted for 1^st^ generation LEX. PK/PD targets for stasis and 1-log kill for EC were 40-50% and 45-75% T >MIC, respectively. Lower targets were noted for 2^nd^ generation CXM. These studies will be integral in evaluation of PK/PD target attainment by integrating human population PK for standard dosing regimens of each drug and MIC distribution data.

**Disclosures:**

**Sujata M. Bhavnani, PharmD; MS; FIDSA**, Adagio Therapeutics, Inc.: Grant/Research Support|Albany Medical Center: Grant/Research Support|Amplyx Pharmaceuticals, Inc.: Grant/Research Support|AN2 Therapeutics: Grant/Research Support|Antabio SAS: Grant/Research Support|Arcutis Biotherapeutics, Inc.: Grant/Research Support|B. Braun Medical Inc.: Grant/Research Support|Basilea Pharmaceutica: Grant/Research Support|BioFire Diagnostics LLC: Grant/Research Support|Boston Pharmaceuticals: Grant/Research Support|Cidara Therapeutics Inc.: Grant/Research Support|Cipla USA: Grant/Research Support|Crestone Inc.: Grant/Research Support|CXC: Grant/Research Support|Debiopharm International SA: Grant/Research Support|Entasis Therapeutics: Grant/Research Support|Genentech: Grant/Research Support|GlaxoSmithKline: Grant/Research Support|Hoffmann-La Roche: Grant/Research Support|ICPD: Ownership Interest|Inotrem: Grant/Research Support|Insmed Inc.: Grant/Research Support|Iterum Therapeutics Limited: Grant/Research Support|Kaizen Bioscience, Co.: Grant/Research Support|KBP Biosciences USA: Grant/Research Support|Matinas Biopharma: Grant/Research Support|Meiji Seika Pharma Co., Ltd.: Grant/Research Support|Melinta Therapeutics: Grant/Research Support|Menarini Ricerche S.p.A.: Grant/Research Support|Mutabilis: Grant/Research Support|Nabriva Therapeutics AG: Grant/Research Support|Paratek Pharmaceuticals, Inc.: Grant/Research Support|Qpex Biopharma: Grant/Research Support|Sfunga Therapeutics: Grant/Research Support|Spero Therapeutics: Grant/Research Support|Suzhou Sinovent Pharmaceuticals Co.: Grant/Research Support|Theravance: Grant/Research Support|tranScrip Partners: Grant/Research Support|University of Wisconsin: Grant/Research Support|Utility Therapeutics: Grant/Research Support|ValanBio Therapeutics Inc.: Grant/Research Support|VenatoRx: Grant/Research Support **Catharine Vincent, Ph.D.**, Adagio Therapeutics: Grant/Research Support|Albany Medical College: Grant/Research Support|Amplyx Pharmaceuticals: Grant/Research Support|AN2: Grant/Research Support|Antabio SAS: Grant/Research Support|Arcutis Biotherapeutics: Grant/Research Support|B. Braun Medical: Grant/Research Support|Basilea: Grant/Research Support|BioFire Diagnostics: Grant/Research Support|Boston Pharmaceuticals: Grant/Research Support|Cidara: Grant/Research Support|Cipla USA: Grant/Research Support|Crestone: Grant/Research Support|CXC: Grant/Research Support|Debiopharma International SA: Grant/Research Support|Entasis: Grant/Research Support|Genentech: Grant/Research Support|GSK: Grant/Research Support|Hoffman-La Roche: Grant/Research Support|Inotrem: Grant/Research Support|Insmed: Grant/Research Support|Iterum Therapeutics: Grant/Research Support|Kaizen Bioscience: Grant/Research Support|KBP Biosciences: Grant/Research Support|Matinas Biopharma: Grant/Research Support|Meiji Seika Pharma: Grant/Research Support|Melinta: Grant/Research Support|Menarini Ricerche: Grant/Research Support|Mutabilis: Grant/Research Support|Nabriva Therapeutics: Grant/Research Support|Paratek Pharmaceuticals: Grant/Research Support|Qpex Biopharma: Grant/Research Support|Sfunga Therapeutics: Grant/Research Support|Spero Therapeutics: Grant/Research Support|Suzhou Sinovent Pharmaceuticals: Grant/Research Support|Theravance: Grant/Research Support|tranScrip Partners: Grant/Research Support|Univ of Wisconsin: Grant/Research Support|Utility Therapeutics: Grant/Research Support|ValanBio Therapeutics: Grant/Research Support|VenatoRx: Grant/Research Support **Brian D. VanScoy, B.S.**, Adagio Therapeutics, Inc.: Grant/Research Support|Albany Medical Center: Grant/Research Support|Amplyx Pharmaceuticals, Inc.: Grant/Research Support|AN2 Therapeutics: Grant/Research Support|Antabio SAS: Grant/Research Support|Arcutis Biotherapeutics, Inc.: Grant/Research Support|B. Braun Medical Inc.: Grant/Research Support|Basilea Pharmaceutica: Grant/Research Support|BioFire Diagnostics LLC: Grant/Research Support|Boston Pharmaceuticals: Grant/Research Support|Cidara Therapeutics Inc.: Grant/Research Support|Cipla USA: Grant/Research Support|Crestone Inc.: Grant/Research Support|CXC: Grant/Research Support|Debiopharm International SA: Grant/Research Support|Entasis Therapeutics: Grant/Research Support|Genentech: Grant/Research Support|GlaxoSmithKline: Grant/Research Support|Hoffmann-La Roche: Grant/Research Support|ICPD: Employee|Inotrem: Grant/Research Support|Insmed Inc.: Grant/Research Support|Iterum Therapeutics Limited: Grant/Research Support|Kaizen Bioscience, Co.: Grant/Research Support|KBP Biosciences USA: Grant/Research Support|Matinas Biopharma: Grant/Research Support|Meiji Seika Pharma Co., Ltd.: Grant/Research Support|Melinta Therapeutics: Grant/Research Support|Menarini Ricerche S.p.A.: Grant/Research Support|Mutabilis: Grant/Research Support|Nabriva Therapeutics AG: Grant/Research Support|Paratek Pharmaceuticals, Inc.: Grant/Research Support|Qpex Biopharma: Grant/Research Support|Sfunga Therapeutics: Grant/Research Support|Spero Therapeutics: Grant/Research Support|Suzhou Sinovent Pharmaceuticals Co.: Grant/Research Support|Theravance: Grant/Research Support|tranScrip Partners: Grant/Research Support|University of Wisconsin: Grant/Research Support|Utility Therapeutics: Grant/Research Support|ValanBio Therapeutics Inc.: Grant/Research Support|VenatoRx: Grant/Research Support **Helio S. Sader, MD, PhD, FIDSA**, AbbVie: Grant/Research Support|Basilea: Grant/Research Support|Cipla: Grant/Research Support|Paratek: Grant/Research Support|Pfizer: Grant/Research Support|Shionogi: Grant/Research Support **David Andes, MD**, Astellas: Advisor/Consultant|Sfunga: Advisor/Consultant

